# The Use of Non-Prescribed Prescription Drugs and Substance Use Among College Students: A 9-Year Follow-Up Cohort Study

**DOI:** 10.3389/fpsyt.2020.00880

**Published:** 2020-08-27

**Authors:** Alicia Busto Miramontes, Lucía Moure-Rodriguez, Ainara Diaz-Geada, Carina Carbia, Fernando Cadaveira, Francisco Caamaño-Isorna

**Affiliations:** ^1^CIBER de Epidemiología y Salud Pública (CIBERESP), Department of Public Health, Universidade de Santiago de Compostela, Santiago de Compostela, Spain; ^2^APC Microbiome Ireland, University College Cork, Cork, Ireland; ^3^Department of Clinical Psychology and Psychobiology, Universidade de Santiago de Compostela, Santiago de Compostela, Spain

**Keywords:** alcohol drinking in college, cannabis, use of non-prescribed prescription drugs, tobacco, young adult

## Abstract

The use of non-prescribed prescription drugs (NPPD) is common in post-modern societies and a significant proportion of youth consume NPPD concomitantly to other drugs. We studied the prevalence of this consumption among university students in Spain, and its relationship to different patterns of alcohol, tobacco, and cannabis use. A cohort study among university students (n=1,380) (2005–2015) was carried out. Students completed self-administered questionnaires at initial (n=1,363, 98.7%), at 2 years (n=875, 75%) and 9 years of follow-up (n=415, 30.5%). Consumption of medicines (last 15 days), risky alcohol consumption (RC), heavy episodic drinking (HED), and tobacco and cannabis use were measured. Multilevel logistic regressions for repeated measures were generated using consumption of medicines with or without medical prescription as dependent variables. Prevalence of RC, HED, tobacco and cannabis had significant reductions during the follow-up. The use of NPPD increased over time, from 35.5% and 33.3% at 18 and 22 years old, respectively, to 49.6% at 27 years old. The highest rates were found among cannabis, tobacco, RC and HED users. For females, cannabis and RC constitute signitifant risk factors for use of NPPD. Conversely, for males, tobacco and cannabis were risk factors for such use of medicines. Later onset of alcohol consumption constitutes a protective factor for females. Our results reveal high prevalence of NPDD among university students. Those who consume NPPD are -at the same time- more likely to be alcohol, tobacco, and cannabis users than those who take medication under prescription. Preventive strategies should be reinforced and focused on this target population to decrease these high levels of poly-consumption.

## Introduction

The use of non-prescribed prescription drugs (NPPD) is becoming an increasing pattern in western societies and it could be understood as a need in the adult population to self-medicate (1). Using medication without a medical prescription is likely to involve some risks for health, and particularly young adults are a sensitive group of users (2–4). According to the European ESPAD Report (2) 9% of adolescents use NPPD concomitantly to other drugs, which appears to be even higher in the United States (5, 6). In Spain, recent reports such as the Spanish National Health Survey (7) shows that 20% of medication for fever, cold, flu and pain is taken without prescription.

Pharmacoepidemiological studies carried out in university populations have shown that the use of medication, with or without medical prescription (5, 8, 9), is not only increasing but also linked to alcohol risky consumption (RC)—understood as that pattern of alcohol use that increases the risk of negative consequences-, heavy episodic drinking (HED)—an alcohol consumption pattern that reaches 0.08 g/dL blood alcohol concentration-, and other drug misuse, as tobacco and cannabis. It is also related with suicidal ideation and suicidal attempts, according to different studies (10–12). In this line, Carrà et al. found a higher probability of non-medical use of prescription pain relievers among respondents who had reported a major depressive episode the previous year, both among minors and adults (13).

Furthermore, HED, RC and cannabis use has been associated with numerous negative consequences social costs for young adults such as increase of traffic accidents (14), more alcohol-related injuries (15), and higher levels of participation in unsafe sex (16). Recently, science literature has started to study the relationships between HED and the use of NPPD in this population (17, 18).

In Spain, several studies have also shown a high prevalence of self-medication among young people (19, 20). Additionally, cross-sectional studies have revealed an association between the consumption of medicines without medical prescription and the consumption of alcohol, tobacco, and cannabis (8, 21, 22).

Our research team has previously studied the Non-Medical Use of Prescription Drugs among University Students, understood as the consumption of certain groups of drugs that have been frequently associated with a more playful intention or at least with non-medical use of this drug (23). In this manuscript we continue to devolve into drug use, although in this case with a different approach and variables, studying the consumption of prescription drugs.

The aim of this study is to determine the prevalence of medicine consumption, both with or without medical prescription, among Spanish university students and its association with the main patterns of alcohol, tobacco, and cannabis use.

## Materials and Methods

### Design, Population, and Sample

We carried out a cohort study among university students (Compostela Cohort 2005, Spain), between November 2005 and February 2015. We used cluster sampling to select the participants. Thus, at least one of the first-year classes was randomly selected from each of the 33 university faculties/schools that conform the college campus of the Universidade de Santiago de Compostela (a total of 53 classes). The Universidade de Santiago de Compostela comprises two campuses: one in the city of Santiago and the other in the city of Lugo. A total of 2,700 students begin each year their college students at this university, but in the day of the collection data only 1,380 were present in class. All students that were present in the class on the day of the survey were invited to participate in the study (n=1.380). This study was approved by the Bioethics Committee of the Universidade de Santiago de Compostela.

During the follow-up, student’s participation decreased, with 1,363 students the first year (98.8%), 875 the second year (75%), and 415 at 9 years (30,5%). The research team made an effort to avoid the decrease in participation, not only by attending classes of 1^st^, 2^nd^, and 3^rd^ year during the second follow-up; by trying to capture students who were left behind in their studies (i.e. not being able to academically progress to the next course); but also by facilitating the schedule that would work better for each participant, including mornings, half days and evenings, as well as weekends in the last follow-up (at 9 years). There were no significant differences among the samples in any key variable. More details about data collection and samples are available in the following reference (24).

### Data Collection Procedures

Researchers visited each first-year classroom in November 2005 and invited all students that were present in the class to participate in the study (1st questionnaire). In November 2007, the same team of researchers visited the third-year classroom (2nd questionnaire). The questionnaires were linked using birth date, sex, school, and class. Students who provided a phone number in the first or second questionnaire were further evaluated by phone at 9-year follow-up (March 2015). On all three occasions, alcohol use was measured with the Galician validated version of the Alcohol Use Disorders Identification Test (AUDIT) (25, 26). In addition, we used another questionnaire that asked about age of onset of alcohol use, tobacco and cannabis consumption and use of prescription drugs. The subjects were asked about their consumption of different medicines, with and without prescription, during the previous 15 days using the Spanish National Health Survey questions regarding this topic (27). The questionnaire was anonymous, the research group assured the participants that the data would only be used for this study and that they had the right to withdraw from the study at any time without consequences.

### Definition of Variables

#### Independent Variables

Heavy episodic drinking (HED). Dichotomous variable generated from the third AUDIT question “How often do you have 6 or more alcoholic drinks per occasion?”, which was coded as follows: never=0, less than once a month=0, once a month=1, once a week=1, daily, or almost daily=1. The sensitivity and specificity of this question with this cut-off value are respectively 0.72 and 0.73, and the area under the curve is 0.767 (95% CI: 0.718–0.816) (28).

Risky consumption (RC). Dichotomous variable generated from AUDIT score (25). A different cut-off value was established according to gender: ≥5 for women; and ≥6 for men. These cut-offs are the ones recommended in the Galician validated version of the AUDIT (26).

Age of onset of alcohol consumption. Four categories were defined for age of onset of alcohol use (after 16 years old, at 16, at 15, before the age of 15).

Cannabis consumption at the beginning of the study and at 2 years of follow-up was measured with the question “Do you consume cannabis when you go out? Never; Sometimes; Most of the times; Always”. These categories were recategorized into No (“never”) and Yes (“sometimes” or “most of the time” or “always”). At 9 years, cannabis consumption was measured using the European Addiction Severity Index (EuropASI) (29).

Tobacco consumption at the beginning of the study and at 2 years of follow-up was also measured as a dichotomous variable: No/Yes. At 9 years of follow-up, we also used the European Addiction Severity Index (EuropASI) (29). Using the EuropASI we also recorded information regarding consumption of cannabis and tobbaco in the last month and lifetime consumption.

#### Dependent Variables

1. Use of prescribed prescription drugs. Dichotomous variable: NO, when the students did not consume any medicine with medical prescription in the previous 15 days; and YES, when they consumed at least one medicine with medical prescription in the previous 15 days. With this variable we measure the consumption of medication that has been pre-written by a physician.
2. Use of non-prescribed prescription drugs (NPPD). Dichotomous variable: NO, when the students did not consume any medicine without medical prescription in the previous 15 days; and YES, when they consumed at least one medicine without medical prescription in the previous 15 days. With this variable, we measure the consumption of medication that has not been prescribed by a physician.

### Statistical Analysis

We used multilevel logistic regression for repeated measures to obtain adjusted Odds Ratios (OR) for the independent variables included in the models. Confidence intervals of 95% (95% CI) were calculated. These models are more flexible than traditional models and therefore allow us to work with correlated data. The same subject was measured several times, and the responses were strongly correlated. The university faculty/school and classroom were random variables. In our data, we have potentially three measures of the same subject (at the ages of 18, 20, and 27). The follow-up time was included as an offset term.

Maximal models were generated, including all theoretical independent variables. From these maximal models, final models were generated. Final models included all significant variables or non-significant variables when their elimination changed the OR of other variables by more than 10%. Data were analyzed using Generalized Linear Mixed Models in SPSS v.20 statistical software.

## Results

Prevalence rates of RC, HED, tobacco, and cannabis consumption at the beginning of the study, and at 2- and 9-year follow-up are shown in **Table 1**. For every substance and pattern of consumption, we can appreciate a significant reduction. There is a trend (not significant) for an increase in RC in both genders and an increase in the practice of HED in men between 18 and 20 years of age. Prevalence of consumption for males remains higher than for females for all drugs, except for tobacco consumption.

**Table 1:** Prevalence rates of alcohol, tobacco, and cannabis consumption at the beginning of the study, and at 2- and 9-year follow-up.

	Percentage (95%CI)					
	Women			Men		
	Initial	2 years	9 years	Initial	2 years	9 years
Risky consumption	51.5	52.2	20.9	58.0	62.2	31.1
Heavy episodic drinking	17.9	16.7	4.9	35.6	43.2	20.0
Cannabis consumption	18.6	16.1	4.0	27.0	19.9	8.9
Tobacco consumption	31.0	19.4	16.9	27.5	19.4	10.1

Proportions of subjects using prescribed and non-prescribed prescription drugs during the previous 15 days are shown in **Table 2**. Females showed a higher use of medication than males throughout the follow-up period. A total of 49.6% of university students consume drugs without medical prescription at the end of the follow-up period, in comparison with 35% who did it at the beginning of the study.

**Table 2:** Proportions of subjects using prescription drugs during the previous 15 days.

	Percentages (%)								
	Subjects using prescribed prescription drugs			Subjects using non-prescribed prescription drugs			Subjects using prescribed and non-prescribed prescription drugs related to the total of subjects using prescription drugs		
	at 18 y	at 20 y	at 27 y	at 18 y	at 20 y	at 27 y	at 18 y	At 20 y	At 27 y
Medication for pain or fever	7.9	6.3	12.0*	15.9	19.0	36.9*	67.2	75.6	76.1
Anxiolytics, sedatives	1.9	1.7	3.4	3.0	2.6	1.0	61.2	60.5	22.2*
Antidepressants, stimulants	0.9	2.4	1.4*	0.5	0.3	0.0	36.8	12.5	0.0*
Medication for colds, flu	12.4	4.7	8.2*	21.7	15.2	15.7*	64.0	77.8	67.0
Antibiotics	11.5	5.7	6.7*	3.1	1.4	0.2*	21.5	19.2	3.4*
Vitamins, minerals, tonics	5.3	2.6	5.3*	6.8	8.1	9.2	56.4	75.5	64.4
Contraceptives^Ť^	12.4	25.9	39.7*	3.2	4.6	6.8*	20.6	15.2	14.7
Any medication^Ŧ^	31.2	27.7	32.5	35.5	33.3	49.6*	61.3	61.9	70.8

^Ť^Out of the total number of female students. ^Ŧ^Contraceptives excluded. *Comparison of proportions, p < 0.05.

**Table 3** presents the proportions of subjects using medical drugs during the previous 15 days in relation to age of onset of use of alcohol, RC, HED, cannabis use, and tobacco use at ages 18, 20, and 27 years. In general, the prevalence of use of NPPD among cannabis, tobacco, RC, and HED users is higher than among non-users. Prevalence among subjects with an early age of alcohol use onset is also higher. There are no significant differences for use of medicines with medical prescription.

**Table 3:** Use of prescription drugs at 18, 20, and 27 years old by profiles of substance use.

Females	Percentage of subjects (%)								
	Use of prescription drugs			Use of prescribed prescription drugs			Use of non-prescribed prescription drugs		
	at 18 y	at 20 y	at 27 y	at 18 y	at 20 y	at 27 y	at 18 y	at 20 y	at 27 y
**Age of onset of use of alcohol**									
After 16 years old	54.9	52.9	70.0	35.4	32.7	35.5	26.8	29.8	42.5
At 16	59.7	51.1	73.3	29.9	27.6	32.7	36.4	30.8	50.5
At 15	67.9	57.3	73.1	36.2	26.7	29.5	43.0	35.1	56.4
Before the age of 15	67.6*	62.9	77.2	39.4	30.5	40.4	45.8*	41.0*	54.4
**Alcohol risky consumption**									
No	58.0	51.9	71.2	34.9	32.0	34.2	31.0	30.3	49.0
Yes	64.4*	58.5	80.9	32.9	28.7	32.4	42.3*	37.2	60.3
**Heavy episodic drinking**									
No	60.2	54.0	72.8	34.5	29.1	33.7	34.6	33.2	51.1
Yes	66.6	61.6	81.2	30.9	30.4	37.5	46.6*	37.5	56.2
**Cannabis consumption**									
No	58.4	55.1	72.1	32.8	30.3	33.0	33.8	33.0	51.3
Yes	74.1	56.5	100*	38.4	24.1	53.8	49.7	38.9*	53.8
**Tobacco consumption**									
No	57.7	55.5	71.1	32.5	28.6	33.7	34.1	34.7	47.8
Yes	68.8*	56.2	83.6*	37.0	32.3	34.5	42.9*	30.8	69.1*
**Total**	61.3	55.3	73.2	33.9	29.3	33.8	36.8	33.9	51.4
**Males**									
**Age of onset of use of alcohol**									
After 16 years old	48.3	41.4	71.4	25.9	10.3	35.7	27.6	34.5	42.9
At 16	50.0	46.5	43.2	28.0	19.7	24.3	33.1	31.0	32.4
At 15	42.0	60.0	68.8	17.4	22.9	31.2	27.5	40.0	56.2
Before the age of 15	56.0	50.0	70.0	25.3	34.2*	30.0	40.4	26.3	50.0
**Alcohol risky consumption**									
No	45.5	41.6	58.1	26.3	19.5	29.0	26.3	29.9	4.9
Yes	50.2	50.4	60.7	21.9	24.8	25.0	36.3*	30.2	46.4
**Heavy episodic drinking**									
No	48.1	48.4	58.3	24.3	25.4	27.8	31.0	30.2	43.1
Yes	48.5	45.0	61.1	22.7	18.8	27.8	34.1	30.0	44.4
**Cannabis consumption**									
No	46.9	42.4	59.8	24.0	20.6	26.8	30.3	26.7	43.9
Yes	52.0	65.9*	50.0	23.0	31.7	37.5	37.0	43.9*	37.5
**Tobacco consumption**									
No	44.6	42.8	58.0	23.4	22.3	25.9	29.7	25.9	43.2
Yes	57.8*	65.0*	66.7	24.5	25.0	44.4	38.2	47.5*	44.4
**Total**	48.2	47.1	58.9	23.7	22.8	27.8	32.1	30.1	43.3

*p < 0.05 among categories.

Finally, multivariate logistic regression models showed that RC (OR=1.35) and cannabis consumption (OR=1.35) are risk factors for NPPD use among women. The results showed that a later age of onset of alcohol use (OR=0.61) constitutes a protective factor. With regard to men, bivariate logistic regression shows that NPPD use is associated with tobacco (OR=1.68) and with cannabis consumption (OR=1.43). (**Table 4**).

**Table 4:** Influence of substance use on the consumption of prescription drugs. Logistic regression.

	Use of prescribed prescription drugs			Use of non-prescribed prescription drugs		
	Women		Men	Women		Men
	Bivariate	Multivariate^Ť^	Bivariate	Bivariate	Multivariate^Ť^	Bivariate
**Age of onset of use of alcohol**						
Before the age of 15	1	1	1	1	1	1
At 15	0.81(0.60–1.10)	0.81(0.60–1.10)	0.65(0.36–1.18)	0.88(0.66–1.18)	0.92(0.68–1.23)	0.92(0.55–1.56)
At 16	0.73(0.55–0.98)	0.73(0.54–0.97)	0.82(0.50–1.34)	0.69(0.52–0.90)	0.65(0.57–0.99)	0.83(0.52–1.32)
After 16 years old	0.92(0.66–1.28)	0.91(0.66–1.27)	0.71(0.39–1.32)	0.51(0.36–0.71)	0.61(0.43–0.83)	0.80(0.46–1.40)
**Alcohol risky consumption**						
No	1		1	1	1	1
Yes	0.91(0.76–1.10)		0.90(0.63–1.29)	1.34(1.12–1.61)	1.35(1.08–1.69)	1.24(0.89–1.72)
**Heavy episodic drinking**						
No	1		1	1		1
Yes	0.94(0.72–1.22)		0.82(0.56–1.21)	1.33(1.04–1.70)		1.04(0.74–1.46)
**Cannabis consumption**						
No	1		1	1	1	1
Yes	1.08(0.84–1.40)		1.17(0.77–1.78)	1.49(1.17–1.91)	1.35(1.03–1.77)	1.43(0.99–2.09)
**Tobacco consumption**						
No	1		1	1		1
Yes	1.21(0.98–1.50)		1.13(0.75–1.72)	1.27(1.03–1.56)		1.61(1.11–1.35)
**Age of the subjects**						
18 years old	1	1	1	1	1	1
20 years old	0.81(0.65–1.00)	0.78(0.62–0.98)	0.95 (0.63–1.43)	0.88 (0.71–1.08)	0.83 (0.67–1.04)	0.91(0.63–1.32)
27 years old	1.00(0.77–1.30)	0.98(0.73–1.30)	1.24 (0.73–2.08)	1.82 (1.41–2.34)	2.04 (1.52–2.73)	1.62(1.01–2.60)

^Ť^Adjusted by the all variables included in the column.

## Discussion

Our findings reveal a very high intake of medicines among university students. This is particularly true for NPPD and this use increases over time. On the contrary, for both patterns of alcohol consumption, as well as for tobacco and cannabis use, we found a significant reduction during the follow-up. The use of NPPD shows a significant association with consumption of alcohol, tobacco, and cannabis. No associations were found for use of medicines with prescription.

The proportion of subjects using NPPD at the end of the 9-years follow-up is higher than initially (10 percentage points). This proportion is significantly higher than the 20% reached for some medicines revealed by the Spanish National Health Survey (7). In line with our results, a high proportion of NPPD has also been found by other studies conducted in similar populations (1, 19, 20). This high prevalence could be partially explained by the period in which data was collected (November 2005, November 2007, and March 2015). A considerable proportion of pharmacists dispense drugs without a prescription in Spain [reaching up to 84.8% for certain types of medications (1, 22). Another potential explanation for the high NPDD in this population, is the typical peak in risky behavior during early adulthood (30), possibly derived from the need of self-affirmation (1) and personal autonomy (31), common characteristics during this period.

Significant differences in the use of medication for pain or fever, anxiolytics and sedatives, antidepressants and stimulants, antibiotics, and contraceptives have been found between the baseline sample and the 9-year follow-up. These differences may be due to the multiple reasons for which these drugs can be prescribed, particularly in the case of fever, pain, colds and flu (7). Although these medications have not euphoria effects and are not used with recreational purposes, this information can help us to describe more accurately the profile of our poli-consumer students to better understand longitudinal changes in their behavior. Regarding anxiolytics and sedatives, the consumption is more prevalent over time, but only for prescribed medication. On the contrary, this use was reduced for medical prescription drugs, for example at the beginning of the study, 61.2% of anxiolytics and sedatives were consumed without medical prescription compared to only 22.2% of consumption without prescription at the end of the follow-up. Similar patterns were found for antidepressants and stimulants. The growing trend of anxiolytic consumption was also found by Fernández-García et al., the authors showed an increase of more than 20% during a similar amount of time (a decade) in Spain (32). Consumption of vitamins, minerals and tonics remained high and stable over the study period. This could be related to an easy accessibility or the common belief that this medication is not real treatment (19, 33). Finally, females present higher prevalence of consumption of medicines in comparison to males. Similar results have been found by other studies, for example Boyd et al. found nearly double rates of medication among high school girls than boys (1, 34–36).

Our results indicate that NPPD use shows a significant association with alcohol, tobacco, and cannabis use. Our results are consistent with those of previous studies that found an association between HED and consumption of medicines without a medical prescription (18) and between cannabis use and non-medical use of prescription drugs (21, 22, 37). In this sense, a study carried out in a national cohort by Carrà et al. showed a high risk of Non-Medical Use of Prescription Pain Relievers only in participants with major depression who had not received treatment in the previous year, which suggests that undiagnosed mental disorders could be affect in these consumption practices (13). Following this line of research, a meta-analysis published in 2019 in cannabis users, indicates that maladaptative behaviors (i.e. coping mechanisms or typical peaks in impulsive behavior) could underly poor health outcomes (such as suicide or in this case NPPD) (35).

After 9 years, cannabis and RC for females and cannabis and tobacco for males still present an association to medicine consumption without prescription. The findings for females are consistent with previous studies that found interaction between being female, RC and use of NPPD (21). Additionally, the latest onset of alcohol use is also a protective factor for the use of medicines without prescription for females, as other investigations also concluded (21). More gender differences were found when considering older students. Females at 27 years old showed a double risk of NPPD, which are consistent with previous investigations (1, 20)

Our study presents some limitations: 1) An analysis per protocol has not been made, which could bias the conclusions of this paper. However, the sample followed is a representative sample of the initial sample, as demonstrated in previous studies published by this research team (23, 24, 36). Therefore, the results are unlikely to be biased by attrition rates; 2) The third question of the AUDIT does not allow to differentiate for gender. Therefore, it is possible that prevalence of HED in women was underestimated by not taking into account women who drink five drinks on a single occasion. However, this only affects descriptive outcomes and not the statistical findings; 3) Questions related to self-reported measures of drugs and medications could underestimate these variables, however this potential underestimation would affect only descriptive results; and 4) Significant brain changes occur between the ages of 18 and 27 which have not been measured and/or discussed in our study.

Our findings reveal a very high intake of medicines among university students, most of them without a medical prescription, a pattern that increases over time. On the contrary, alcohol, tobacco and cannabis consumption decrease with age. Consumption of medicines without prescription shows a significant association with consumption of alcohol, tobacco and cannabis. This consumption of medicines might be another—understudied—form of poly-consumption of drugs (6). Further studies are needed to clarify questions related to the influence of parental and peers use of medication without medical prescription and co-occurring mental health problems. In light of these results, it is necessary to create further preventive campaigns for students on use of NPPD and concomitant drug consumption.

## Data Availability Statement

The datasets presented in this study can be found in online repositories. The names of the repository/repositories and accession number(s) can be found below: Epidemiology and Public Health Group. Data from: The use of non-prescribed prescription drugs and substance use among college students: a 9-years follow-up cohort study. USC Digital Repository. (2020) https://www.usc.gal/saudep/publicacion/the-use-of-non-prescribed/.

## Ethics Statement

The studies involving human participants were reviewed and approved by Bioethics Committee of the Universidade de Santiago de Compostela. The patients/participants provided their written informed consent to participate in this study.

## Author Contributions

FC-I and FC have designed the study. ABM, FC-I, LM-R, AD-G, and CC have analyzed and interpreted data. ABM, LM-R, and FC-I have written the article. All authors contributed to the article and approved the submitted version.

## Funding

Funding for this study was provided by the Plan Nacional sobre Drogas (Spain) (2005/PN014) and the Fondo de Investigación Sanitaria (Spain) (PI15/00165). CC has been funded by the European Union’s Horizon 2020 research and innovation program with the Marie Sklodowska-Curie grant No. 754535.

## Conflict of Interest

The authors declare that the research was conducted in the absence of any commercial or financial relationships that could be construed as a potential conflict of interest.
